# A novel deep learning conditional generative adversarial network for producing angiography images from retinal fundus photographs

**DOI:** 10.1038/s41598-020-78696-2

**Published:** 2020-12-09

**Authors:** Alireza Tavakkoli, Sharif Amit Kamran, Khondker Fariha Hossain, Stewart Lee Zuckerbrod

**Affiliations:** 1 Department of Computer Science and Engineering, University of Nevada, Reno, Reno, NV 89557 USA; 2 Department of Computer Science, Deakin University, Melbourne, VIC 3217 Australia; 3 Department of Ophthalmology, Houston Eye Associates, Houston, TX 77401 USA

**Keywords:** Image processing, Machine learning, Software, Computer science

## Abstract

Fluorescein angiography (FA) is a procedure used to image the vascular structure of the retina and requires the insertion of an exogenous dye with potential adverse side effects. Currently, there is only one alternative non-invasive system based on Optical coherence tomography (OCT) technology, called OCT angiography (OCTA), capable of visualizing retina vasculature. However, due to its cost and limited view, OCTA technology is not widely used. Retinal fundus photography is a safe imaging technique used for capturing the overall structure of the retina. In order to visualize retinal vasculature without the need for FA and in a cost-effective, non-invasive, and accurate manner, we propose a deep learning conditional generative adversarial network (GAN) capable of producing FA images from fundus photographs. The proposed GAN produces anatomically accurate angiograms, with similar fidelity to FA images, and significantly outperforms two other state-of-the-art generative algorithms ($p<.001$ and $p<.0001$). Furthermore, evaluations by experts shows that our proposed model produces such high quality FA images that are indistinguishable from real angiograms. Our model as the first application of artificial intelligence and deep learning to medical image translation, by employing a theoretical framework capable of establishing a shared feature-space between two domains (i.e. funduscopy and fluorescein angiography) provides an unrivaled way for the translation of images from one domain to the other.

## Introduction

For a long time fluorescein angiography (FA) combined with Retinal Funduscopy have been used for diagnosing retinal vascular and pigment epithelial-choroidal diseases^1^. The process requires the injection of a fluorescent dye which, depending on the age and cardiovascular structure of the eye, appears in the optic vascular system within 8–12 s and can stay up to 10 min^2^. Although generally considered safe, there have been reports of mild to severe complications due to allergic reactions to the dye^3–5^. Side effects can range from nausea and heart attack, to anaphylactic shock and death^6–10^. In addition, leakage of fluorescein at the injection site can occur.

Given the complications and the risks associated with this procedure, non-invasive, affordable, and computationally effective alternatives to FA are quite necessary. The only current alternative to fluorescein angigraphy (FA) for visualizing retinal vasculature is carried out by additional hardware and software modifications to Optical Coherence Tomography (OCT)^11,12^, called OCT Angiography (OCTA)^13,14^. Despite the ability to generate visual blood flow maps without the adverse side effects of FA, OCTA systems are not widespread in assessment of retinal vascular diseases, due to their cost, the need for multiple acquisitions in the same anatomical location^15^, and limited field of view (FOV). In addition, the recent CoVID-19 pandemic has had a significant negative impact on ophthalmologists’ ability to conduct in-clinic exams^16^, demonstrated the limitations of the current state of tele-ophthalmology^17^, and highlighted the need for developing effective, low-cost, and reliable alternatives for both in-home and in-clinic measurements.

The introduction of convolutional neural networks and a gradient-based optimization regime for training these networks by LeCun et al.^18^ has resulted in a subsequent deep learning revolution^19^ in the field of Artificial Intelligence (AI). Not only has deep learning significantly improved the performance of visual object classification^20^, object detection^21^, semantic^22^ and instance segmentation^23^, and de-noising algorithms^24^, but it also has introduced novel computational frameworks such as super-resolution^25^, image generation^26^, and style-transfer^27,28^. Despite its late arrival to the field of ophthalmology compared to other medical domains^29,30^, deep learning has already started to play a transformative role in ophthalmology ranging from noise removal^31^, to disease classification^32–34^, to disease marker segmentation^33,35,36^. These advances resulted in the first automatic AI-enabled Diabetic Retinopathy (DR) system, called IDx-DR^37^, to be approved by the FDA in 2018^38^. Although these AI-inspired ophthalmic systems have produced reasonable results^39–41^, they only utilize basic and rudimentary deep learning architectures, susceptible to data bias^42^ and the need for massive amount of training samples^43^.

In order to address the inefficiency in utilizing generic deep learning models in ophthalmology, our team has developed effective architectures^44–46^ for retinal disease diagnosis. Our proposed architectures are capable of mapping ophthalmic images (e.g. fundus, OCT, angiograms, etc.) onto a high dimensional feature space (i.e. a latent manifold), rich with clustered information pertaining to the ocular anatomical structures. These latent representations of anatomical structures will have the potential to advance the current state of deep learning in ophthalmology. Specifically, the ability to map ocular structures from different imaging modalities such as funduscopy, FA, and OCT into a shared latent manifold will unlock novel approaches to fusing the information acquired from these modalities to enable far more useful information for disease diagnosis and prognosis. This is the premise of the proposed work in establishing a relationship between FA and fundus images in their shared latent manifold for the purpose of generating anatomically accurate FA images from fundus photographs. To our knowledge, and unlike the image generation method proposed by Lee et al.^47^, the work proposed in this paper is the first deep learning application in ophthalmic imaging to generate images from truly different modalities.

Lee et al. have leveraged the relationship between the OCT and OCTA data to generate OCTA-like images solely from OCT images^47^. This technique is was the first AI model to explore ophthalmic applications of deep learning beyond classification and segmentation. However, this architecture has several limitations that prevent it from performing as an effective domain transfer system (i.e. generating images from inherently different data modalities). First, the input domain (OCT) and the output domain (OCTA) are significantly correlated and do not constitute a truly different anatomical structure modalities. Second, the deep learning module used in this approach is a simple autoencoder^48^ adapted from an encoder/decoder architecture for segmentation^49^. As such, this network is not capable of exploiting the significantly different probability distributions governing the input and output modalities for the purpose of generating real OCTA images from OCT input data. Finally, although OCT technology is more widely used and less expensive than OCTA, OCT imaging is still expensive and requires clinic visits, preventing truly ubiquitous use as an in-home alternative.

Given the ability to use our previous deep learning architectures^44–46^ to produce feature rich latent manifolds from input ocular structural images, we sought to explore the ability to map paired fundus photographs and FA images onto a shared latent manifold in which the retina vasculature from both domains share similar feature representations. This approach has its roots in the recently introduced Generative Adversarial Networks (GANs)^27,50–52^ in the field of deep learning. Although GANs have been recently utilized in the field of ophthalmology from predicting post-therapeutic OCT images^53^, to removing shadows from OCT images^54^, these studies primarily focus on a single domain modality. The proposed study is the first of its kind to demonstrate the viability of cross-modality transformation in the field of ophthalmic imaging. Comparisons of our model with state-of-the-art image generation and style transfer systems showed that our model outperforms these networks, both qualitatively and quantitatively. In addition, expert ophthalmologists were asked to distinguish from a random set of balanced real FA images and those angiograms generated by our model in two trials. Results show that the angiograms generated by the proposed network are quite indistinguishable from real FA images.

It is worth discussing that the proposed study is designed as a proof-of-concept framework to demonstrate the technical and computational viability of performing image domain transformation to provide adjunctive information in the absence of FA modalities. As such, this framework is a part of an evolutionary study to establishing shared manifolds between different imaging modes that can be utilized to improve the diagnostic capabilities in the absence of a comprehensive battery of tests.

**Figure 1:**
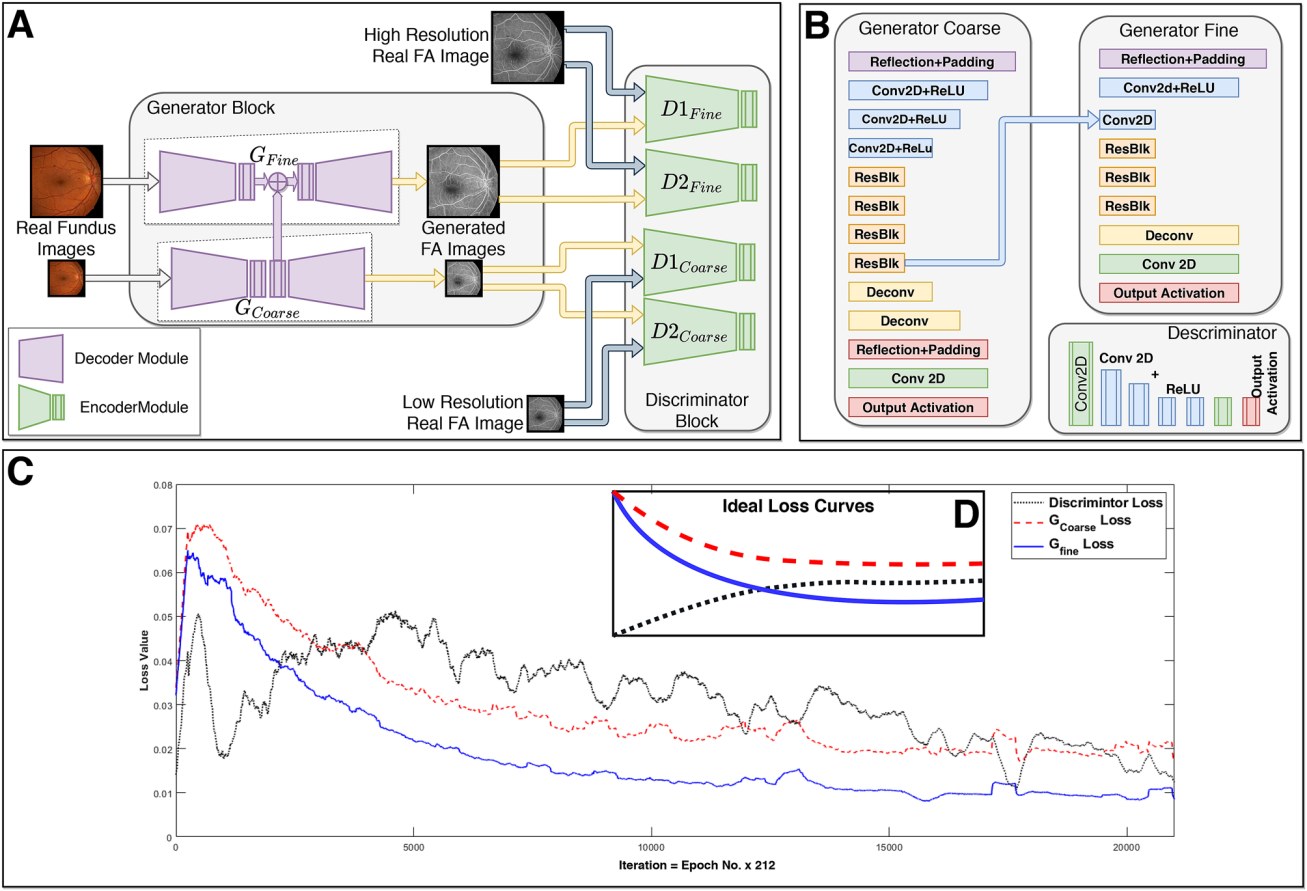
Generative Adversarial Network (GAN) models used for producing anatomically accurate FA images from fundus photographs (**A**). Details of the generator blocks and discriminator blocks of the proposed network (**B)**. Generator and discriminator block loss values as a function of training iterations (**C**). Ideal loss curves (**D**).

## Results

We designed a conditional generative adversarial network (GAN) comprising of two generator modules and four discriminator modules (Fig. 1A) to take fundus photographs and produce anatomically accurate FA images inferred from the fundus images. The generator block consists of two generator modules, the fine and coarse generators, which are designed in a U-shaped encoder-decoder manner. The coarse generator is comprised of a reflection+padding block, three convolution (Conv)+batch normalization (BN)+leaky rectified linear units (ReLU), and four novel residual blocks^44,45^ (ResBlk), followed by two transpose convolution (Deconv), one reflection+padding, one Conv, and an output activation layers (Fig. 1B-left), and is responsible for generating coarse and global structures of the FA image such as the structures of the macula, optic disc, color, contrast, and brightness. The fine generator is comprised of one reflection+padding, one Conv+BN+ReLU, and one Conv layer, followed by three ResBlk, one Deconv, one Conv, and one output activation layer (Fig. 1B-right), and produces local information including retinal venules, arterioles, hemorrhages, exudates, and microaneurysms. The last ResBlk of the coarse generator is added to the first Conv layer of the fine generator to integrate the global features from the coarse generator with the local information in the fine generator. The discriminator blocks of the proposed network are encoders tied to a final layer of fully connected binary classification, and takes a pair of real and generated FA images and decide which one is real. The fine discriminators take the pair of real and generated images at full resolution, while the coarse discriminators take images at half resolution (Fig. 1A). Each discriminator is comprised of an initial Conv layer and Conv+BN+ReLU layers, followed by one last Conv layer and finally the output activation layer (Fig. 1B-bottom). In our implementation the fine generator has 170,305 trainable parameters, the coarse Generator has 6,695,041, and each discriminator has 234,785 trainable parameters for a total of around 7.8 million parameters.

The models were trained over 100 epochs, with each epoch comprising of 212 iterations, in a minimax setup in which the generators attempt to produce realistic FA images and discriminators attempt to correctly identify whether a given FA image is real or produced by the generators. The loss values for the coarse and fine generators as well as the combined loss values of the discriminator block are shown in Fig. 1C. The black dotted curve is the discriminator loss, while the solid blue and dashed red curves are the fine and course generator losses, respectively. At the beginning of the training, the generators produce sampled random images from a latent representation of FA images, and thus the discriminators can easily identify real from generated FA images. This can be observed as the smaller (better) discriminator loss values compared to generator loss values for early epochs in Fig. 1C. As training progresses, the generators learn to produce more realistic FA images which become increasingly difficult for discriminators to identify as not real, as observed from the downward trend in generator loss and upward trend in discriminator loss curves in early epochs in Fig. 1C. The goal of the network is to reach an equilibrium where the loss values for the generators and discriminators stabilize (late epochs in Fig. 1C). The ideal loss curves for a generative adversarial network (GAN) is shown in Fig. 1D, in which the network reaches the Nash equilibrium.

For training, we use the fundus and angiography data-set provided by Hajeb et al.^55^. The data-set includes 30 pairs of diabetic retinopathy and 29 pairs of normal FA and fundus images from 59 patients. Fundus photographs are in color format, whereas angiograms are in gray-scale format. Our proposed network is capable of performing with high degrees of accuracy even on this small dataset. To improve the accuracy of training we perform a randomized data augmentation process by which *N* random crops of size $512\times 512$ from each images are extracted. These random images are then processed through geometric and photometric manipulations and then used for training the model. So, the total number of training sample is $O(17\times N\times f)$, where *f* is the number of photometric and geometric manipulations. This process can potentially generate an infinite number of training samples for our deep learning algorithm, addressing data limitation issues in deep learning. For example, with $N=500$ crops from 17 images and with 10 geometric and photometric manipulations, the training set will consist of 85,000 pairs of fundus photographs and FA images. In our method we used image rotation, horizontal flip, and vertical flip as geometric transformations. Photometric transformations used in the proposed data augmentations include gamma correction, contrast stretching, contrast compression, and color manipulations.

**Figure 2:**
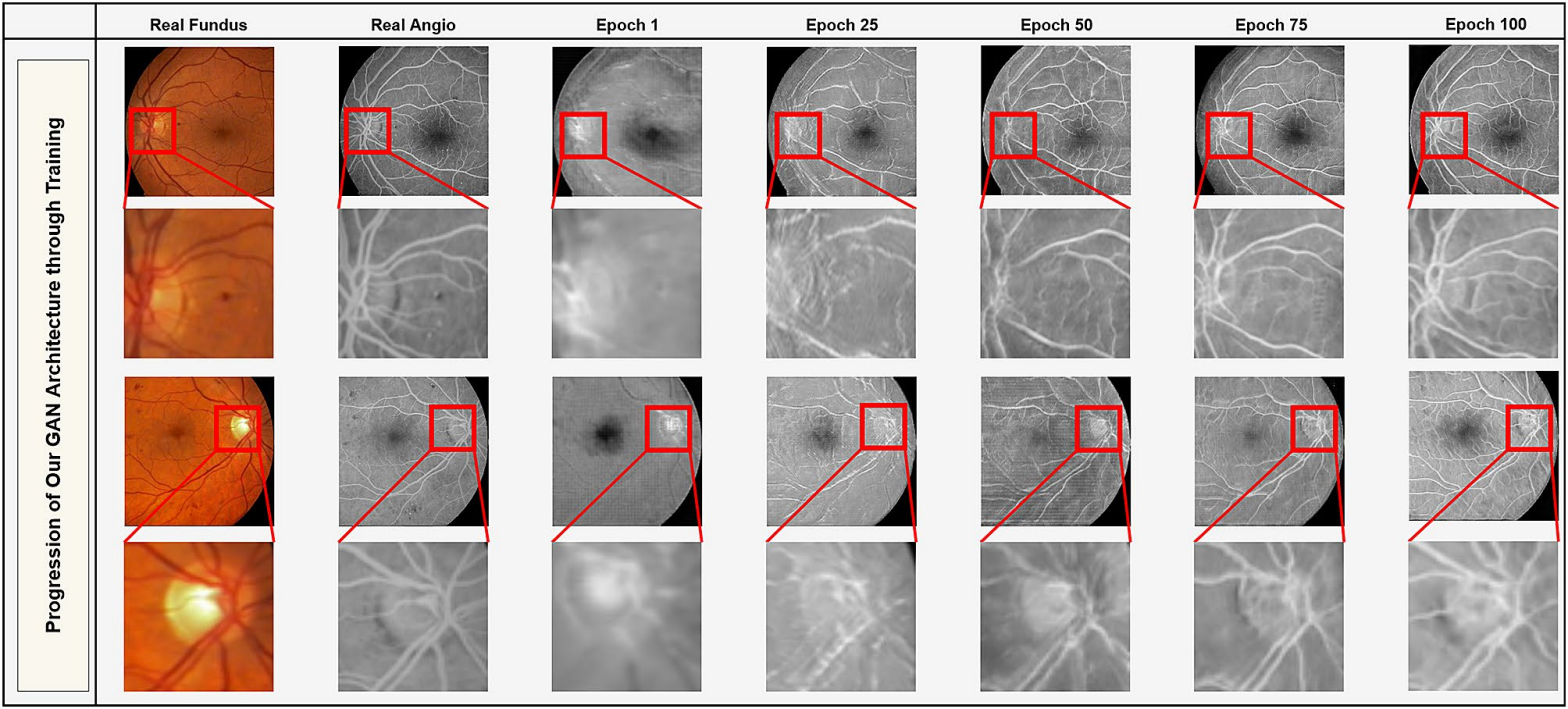
Our proposed method generates more realistic FA images after each epoch as training progresses.

The results of the training is shown in Fig. 2 over the course of 100 epochs. In this figure, the first and the third rows are the original fundus images, their paired FA image, and the generated results after training at epochs 1, 25, 50, 75, and 100, respectively. The second and fourth rows show the magnification of the red rectangular regions for a better visual representation of the vascular structure generated by our method as the training progresses. Our proposed method learns the global information like optic-disc, fovea position, and large vascular structures first. Next, it tries to learn the minuscule vascular structures, e.g., arteries and veins in a progressive manner.

**Figure 3:**
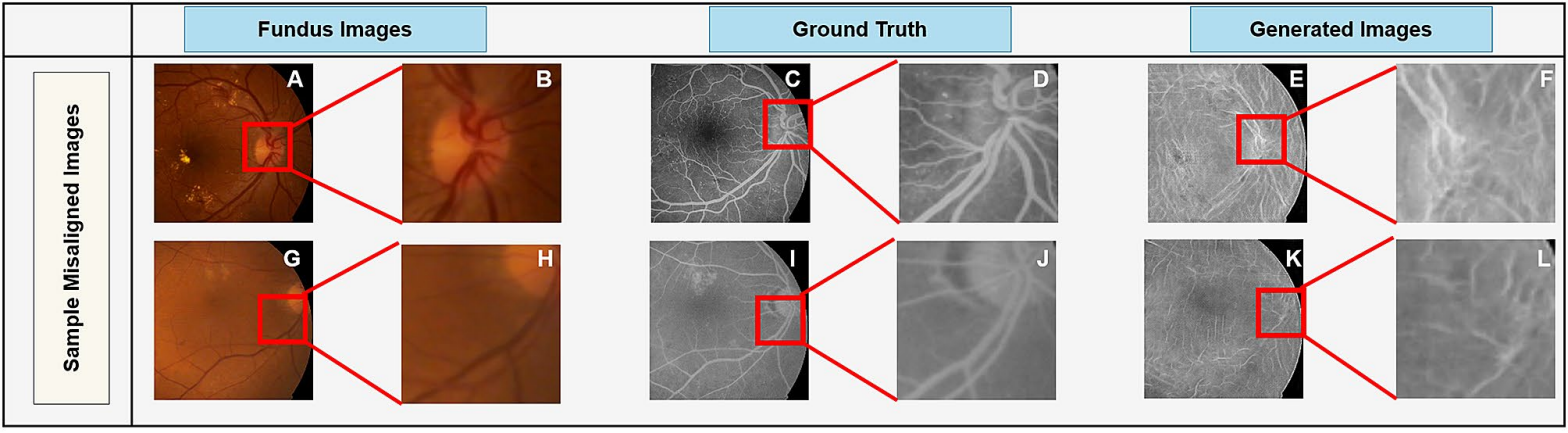
Results of training on unaligned pairs of fundus and angio images. Original fundus images (**A**,**G**) are not aligned with the FA images (**C**,**I**), and as a result the generated images (**E**,**K**) are deteriorated. (**B**,**D**,**F**,**H**,**J**, and **L** are the magnified views of red boxed regions in **A**,**C**,**E**,**G**, **I**, and **K**, respectively.

### Fundus-Angio alignment effects

Training on unaligned images hampers the synthesizing of realistic FA images. Although the global information such as the overall intensity, contrast, and the location of geometric features such as the optic-disc are retained, local information like vascular structures are distorted or absent from the generated image (Fig. 3). Without proper alignment of the paired fundus and FA images used for training, the deep learning GAN architectures fail to generate accurate vascular structures. In order to address this problem, there is a need for aligning the FA images with their fundus counterparts prior to training. The algorithm 1 shows the process by which a misaligned FA image could be aligned with its fundus counterpart. The process takes as input a pair of fundus and FA images and using a fast SIFT feature extractor, called SURF^56^, finds corresponding features between the two images. Singular valued decomposition technique will be then utilized on the matched features between the fundus and FA images to uncover the transformation ($\Phi$) between the two images. This transformation will be then utilized to align the FA image with the fundus photograph.

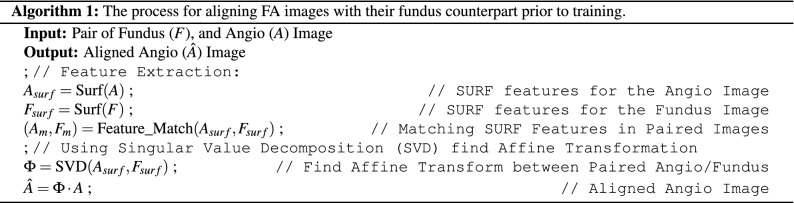



**Figure 4:**
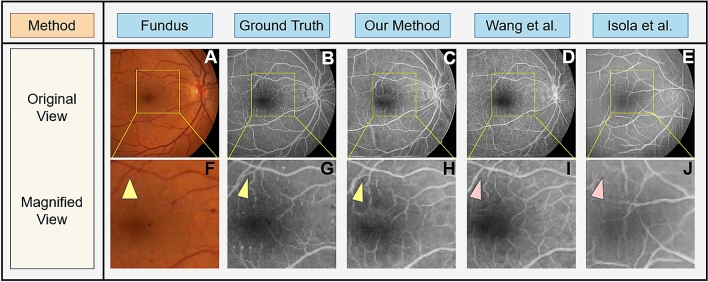
Paired fundus photographs (**A**) and ground truth FA images (**B**). FA images generated by our proposed method (**C)** and those generated by traditional GANs proposed by Wang et al. (**D**)^50^ and Isola et al. (**E**)^27^. Subfigures (**F–J**) show magnification of the yellow boxed areas from (**A–E**). Microvascular structures are not quite visible in the fundus photographs (**A,F**), but are visible in the original FA images (**B,G**) pointed to by the yellow triangles. Our deep learning GAN is capable of producing these microvascular structures pointed to by the yellow triangle, as well as macro vasular structures (**C,H**). The GAN proposed by Wang et al.^50^ misses these microvascular structures (**D,I**) while GAN proposed by Isola et al.^27^ is not able to produce any reliable vascular structure (**E,J**)—see red triangles.

**Figure 5:**
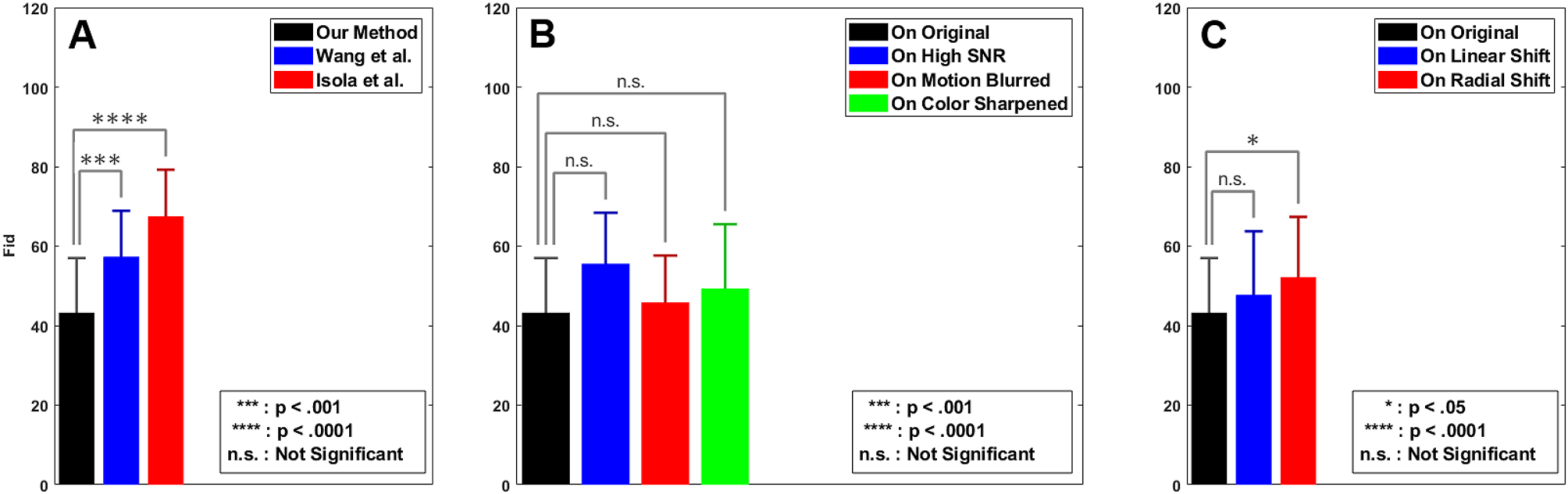
Comparison of the proposed deep learning FA generation model with the other GANs and under different conditions. (**A**) The FIDs for images generated by the proposed method compared with that of Wang et al.^50^ and Isola et al.^27^—lower FID measures represent more accuracy. (**B**) FID measure of the images generated by the proposed on input fundus photographs taken under varying conditions—high signal to noise ratio (SNR), motion blur, and color sharpnening. (**C**) FID measure of the images generated by the proposed on input fundus photographs with slight anatomical alterations—translational vascular shift and rotational vascular shift.

### FA image generation

The first experiment was designed to establish the performance of our proposed method in generating anatomically accurate FA images from fundus photographs, and compare our results with the leading conditional generative adversarial networks proposed by Wang et al.^50^ and Isola et al.^27^ Fig. 4 shows the results of this comparison. In this experiment we supplied the trained networks with a fundus image (Fig. 4A) while the paired FA image of the same patient (Fig. 4B) was held-out as ground truth. Figure 4C–E show the generated FA images by our proposed algorithm, the Wang et al. method^50^, and the Isola et al. method^27^, respectively. The yellow rectangular regions of Fig. 4A–E are magnified and shown in Fig. 4F–J for comparison purposes. Our FA generated images (Fig. 4H) have very good quality and are anatomically accurate compared to the held-out FA images (Fig. 4G) as details and even small vascular structures, pointed by yellow triangles, are preserved and generated compared to the other two methods, pointed by red triangles (Fig. 4I,J).

**Table 1:** Comparisons between Fréchet inception distance (FID) Achieved by Our Method Compared with Wang et al. and Isola et al.

	Generated	Motion blurred	Sharpened	Noise	Linear shift	Radial shift
Ours	43	45	51	49	47	53
Wang et al.^50^	57 ($\uparrow 14$)	61 ($\uparrow 16$)	64 ($\uparrow 13$)	63 ($\uparrow 14$)	62 ($\uparrow 15$)	64 ($\uparrow 11$)
Isola et al.^27^	68 ($\uparrow 25$)	64 ($\uparrow 21$)	67 ($\uparrow 16$)	62 ($\uparrow 13$)	61 ($\uparrow 14$)	63 ($\uparrow 10$)

For quantitative evaluation we use two established measures, i.e., the Fréchet inception distance (FID)^57^ and structural similarity measures (SSIM)^58^. FID is a metric that measures the distance between feature vectors calculated for real and generated images. Since FID represents a distance metric, lower FID measures mean higher accuracy in generating images. FID allows for comparing how accurately the generated FA images represent anatomical features compared to the ground truth. Comparisons of FID measures between our proposed method and those presented by Wang et al.^50^ and Isola et al.^27^ show that our method produces significantly more accurate FA images, $p=.0005$ and $p=4.5\times 10^{-6}$, respectively (Fig. 5A).

Table 1 shows the average FID values of our proposed method compared to those of Wang et al.^50^ and Isola et al.^27^. The lower the FID the better the generated image. As it can be seen our method produces consistently lower FID measures when producing FA images from the original fundus image and from its transformed counterparts when motion blur, sharpening, noise, linear shift, and radial shifts are applied.

The SSIM is a well-known quality metric used to measure the similarity between two images. It is considered to be correlated with the quality perception of the human visual system (HVS) and is designed by modeling any image distortion as a combination of three factors that are loss of correlation, luminance distortion, and contrast distortion^59^. Comparisons of SSIM measures between our proposed method and those presented by Wang et al.^50^ ($p<.0003$) and Isola et al.^27^ ($p<3\times 10^{-7}$) show that our method produces significantly more accurate FA images compared to both.

**Table 2:** Comparisons between structural similarity measure (SSIM) achieved by our method compared with Wang et al.^50^ and Isola et al.^27^.

	Generated	Motion blurred	Sharpened	Noise	Linear shift	Radial shift
Ours	0.67	0.63	0.61	0.64	0.67	0.64
Wang et al.^50^	0.58 ($\downarrow 0.09$)	0.58 ($\downarrow 0.05$)	0.50 ($\downarrow 0.12$)	0.56 ($\downarrow 0.08$)	0.53 ($\downarrow 0.13$)	0.49 ($\downarrow 0.15$)
Isola et al.^27^	0.50 ($\downarrow 0.17$)	0.45 ($\downarrow 0.17$)	0.47 ($\downarrow 0.14$)	0.46 ($\downarrow 0.18$)	0.50 ($\downarrow 0.17$)	0.50 ($\downarrow 0.17$)

Table 2 shows the average SSIM values of our proposed method compared to those of Wang et al.^50^ and Isola et al.^27^. The higher the SSIM the better the generated image. As it can be seen our method produces consistently higher SSIM measures when producing FA images from the original fundus image and from its transformed counterparts when motion blur, sharpening, noise, linear shift, and radial shifts are applied.

#### Results on changes to fundus image acquisition

**Figure 6:**
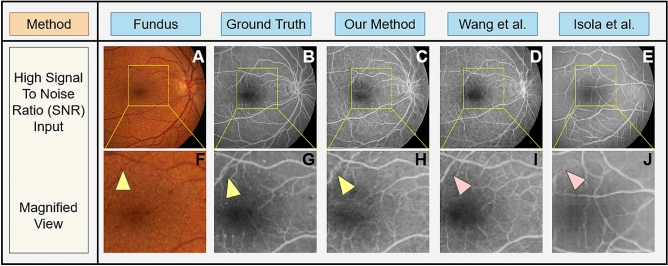
Paired fundus photographs (**A**) acquired under high signal to noise ration (SNR) and ground truth FA images (**B**). FA images generated by our proposed method (**C)** and those generated by traditional GANs proposed by Wang et al. (**D**)^50^ and Isola et al. (**E**)^27^. Subfigures (**F–J**) show magnification of the yellow boxed areas from (**A–E**). Microvascular structures are not quite visible in the fundus photographs (**A,F**), but are visible in the original FA images (**B,G**) pointed to by the yellow triangles. Our deep learning GAN is capable of producing these microvascular structures pointed to by the yellow triangle, as well as macro vasular structures (**C,H**). The GAN proposed by Wang et al.^50^ misses these microvascular structures (**D,I**) while GAN proposed by Isola et al.^27^ is not able to produce any reliable vascular structure (**E,J**)—see red triangles.

An important benefit of the proposed method is the robustness in accuracy of generated FA images from fudus photographs subject to varying imaging issues such as high signal to noise ratio (SNR), motion blur, and color sharpness. Figure 6 shows the generated FA images from noisy fundus photographs (Fig. 6A) compared to the FA image of same subject (Fig. 6B). Using a high SNR fundus photograph as input, Fig. 6C–E show FA images produced by our proposed algorithm, the Wang et al. method^50^, and the Isola et al. method^27^, respectively. The yellow rectangular regions of Fig. 6A–E are magnified and shown in Fig. 6F–J for better viewing. As shown by the yellow triangle in Fig. 6H, small vasculature are preserved and generated by our method, but they are lost in the generated FA images by Wang el al.^50^ and Isola et al.^27^—red triangles in Fig. 6I,J. Comparisons on the FA image generated by our proposed method from normal and from high SNR images show no statistically significant difference in FID measures (Fig. 5B).

**Figure 7:**
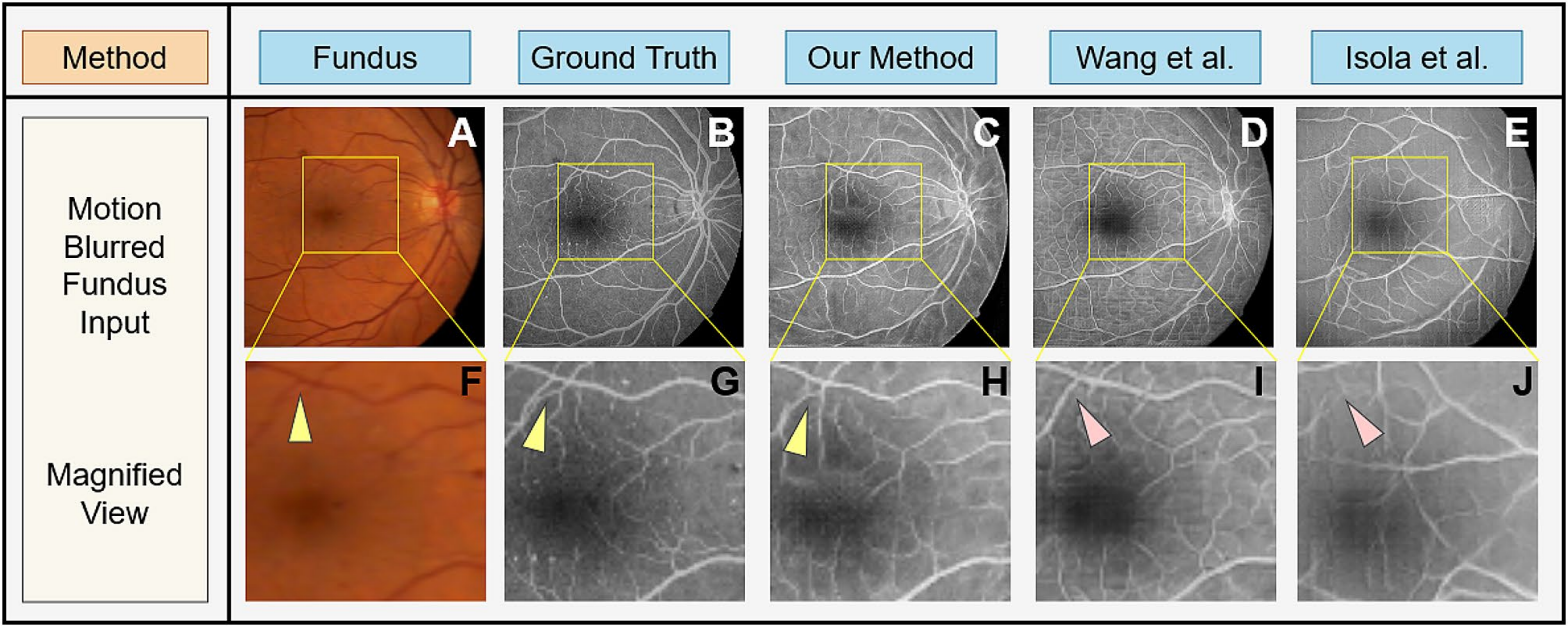
Paired fundus photographs (**A**) acquired under motion blur and ground truth FA images (**B**). FA images generated by our proposed method (**C)** and those generated by traditional GANs proposed by Wang et al. (**D**)^50^ and Isola et al. (**E**)^27^. Subfigures (**F–J**) show magnification of the yellow boxed areas from (**A–E**). Microvascular structures are not quite visible in the fundus photographs (**A,F**), but are visible in the original FA images (**B,G**) pointed to by the yellow triangles. Our deep learning GAN is capable of producing these microvascular structures pointed to by the yellow triangle, as well as macro vasular structures (**C,H**). The GAN proposed by Wang et al.^50^ misses these microvascular structures (**D,I**) while GAN proposed by Isola et al.^27^ is not able to produce any reliable vascular structure (**E,J**)—red triangles.

Figure 7 shows the generated FA images from motion blurred fundus photographs (Fig. 7A) compared to the FA image of same subject (Fig. 7B). Using a motion blurred fundus photograph as input, Fig. 7C–E show FA images produced by our proposed algorithm, the Wang et al. method^50^, and the Isola et al. method^27^, respectively. The yellow rectangular regions of Fig. 7A–E are magnified and shown in Fig. 7F–J for better viewing. As shown by the yellow triangle in Fig. 7H, small vasculature are preserved and generated by our method, but they are lost in the generated FA images by Wang el al.^50^ and Isola et al.^27^—red triangles in Fig. 7I,J. Comparisons on the FA image generated by our proposed method from normal and from motion blurred images show no statistically significant difference in FID measures (Fig. 5B).

**Figure 8:**
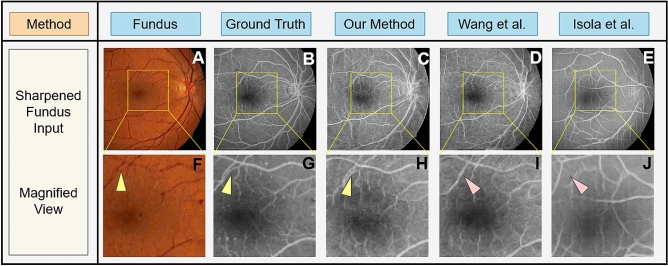
Paired color sharpened fundus photographs (**A**) and ground truth FA images (**B**). FA images generated by our proposed method (**C)** and those generated by traditional GANs proposed by Wang et al. (**D**)^50^ and Isola et al. (**E**)^27^. Subfigures (**F–J**) show magnification of the yellow boxed areas from (**A–E**). Microvascular structures are not quite visible in the fundus photographs (**A,F**), but are visible in the original FA images (**B,G**) pointed to by the yellow triangles. Our deep learning GAN is capable of producing these microvascular structures pointed to by the yellow triangle, as well as macro vascular structures (**C,H**). The GAN proposed by Wang et al.^50^ misses these microvascular structures (**D,I**) while GAN proposed by Isola et al.^27^ is not able to produce any reliable vascular structure (**E,J**)—see red triangles.

Figure 8 shows the generated FA images from fundus photographs subject to color and contrast sharpness (Fig. 8A) compared to the FA image of same subject (Fig. 8B). Using a sharpened fundus photograph as input, Fig. 8C–E show FA images produced by our proposed algorithm, the Wang et al. method^50^, and the Isola et al. method^27^, respectively. The yellow rectangular regions of Fig. 8A–E are magnified and shown in Fig. 8F–J for better viewing. As shown by the yellow triangle in Fig. 8H, small vasculature are preserved and generated by our method, but they are lost in the generated FA images by Wang el al.^50^ and Isola et al.^27^—red triangles in Fig. 8I,J. Comparisons on the FA image generated by our proposed method from normal and from sharpened images show no statistically significant difference in FID measures (Fig. 5B).

**Figure 9:**
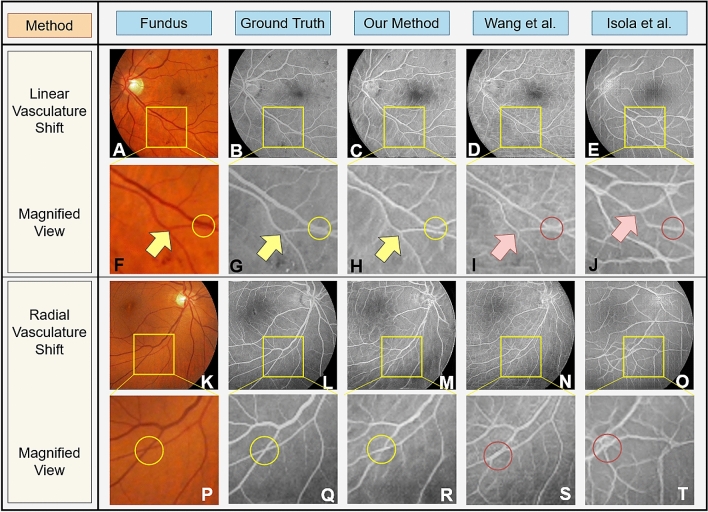
Paired color fundus photograph when vascular structure has small shifts (**A**) or under large vascular movements (**K**) and ground truth FA images (**B,L**). FA images generated by our proposed method (**C**) and those generated by traditional GANs proposed by Wang et al. (**D,N**)^50^ and Isola et al. (**E,O**)^27^. Subfigures (**F–J** and **P–T**) show magnification of the yellow boxed areas from (**A–E** and **K–O**). Our deep learning GAN is capable of accurately producing the changes in vascular structures pointed to by the yellow triangle and circles (**H,R**). The GAN proposed by Wang et al.^50^ misses some of these structural changes (**I,S**) while the network proposed by Isola et al.^27^ is not able to produce any reliable vascular structure (**J,T**)—see red triangles and circles.

### Results on anatomical structure changes

Although robustness to fuduscopy imaging variations should not impact the results of the FA image generation, certain anatomical changes to the vascular structure should be identified and utilized in the image generation process. Our proposed approach has the capability of generating anatomically correct FA images from fundus photographs that contain two kinds of anatomical changes, i.e. slight linear (translational) shift in the vascular pattern and radial shifts changing the curvature of blood vessels. Figure 9 shows the generated FA images from fundus photographs subject to anatomical changes to the structure of retina blood vessels (Fig. 9A,K) compared to the FA image of same subject (Fig. 9B,L). Figure 9C–E show FA images produced by our proposed algorithm, the Wang et al. method^50^, and the Isola et al. method^27^, respectively, on a fundus photograph containing slight blood vessel shift. The yellow rectangular regions of Fig. 9A–E are magnified and shown in Fig. 9F–J for better viewing. As shown by the yellow triangle and circle in Fig. 9H, small vasculature are preserved and generated and the slight vascular shifts are reconstructed with high fidelity by our method, but they are lost in the generated FA images by Wang el al.^50^ and Isola et al.^27^—red triangles in Fig. 9I,J. Comparisons on the FA image generated by our proposed method from normal and from the linear vasculature shifts show no statistically significant difference in FID measures (Fig. 5C).

Figure 9M–O show FA images produced by our proposed algorithm, the Wang et al. method^50^, and the Isola et al. method^27^, respectively, on a fundus photograph containing radial blood vessel shifts. The yellow rectangular regions of Fig. 9K–O are magnified and shown in Fig. 9P–T for better viewing. As shown by the yellow circle in Fig. 9R, the distortions in the blood vessel patterns are reconstructed with high fidelity by our method, but they are lost in the generated FA images by Wang el al.^50^ and Isola et al.^27^—red triangles in Fig. 9S,T. Comparisons on the FA image generated by our proposed method from normal and from the linear vasculature shifts show a statistically significant difference ($p=.010$) in FID measures (Fig. 5C).

**Figure 10:**
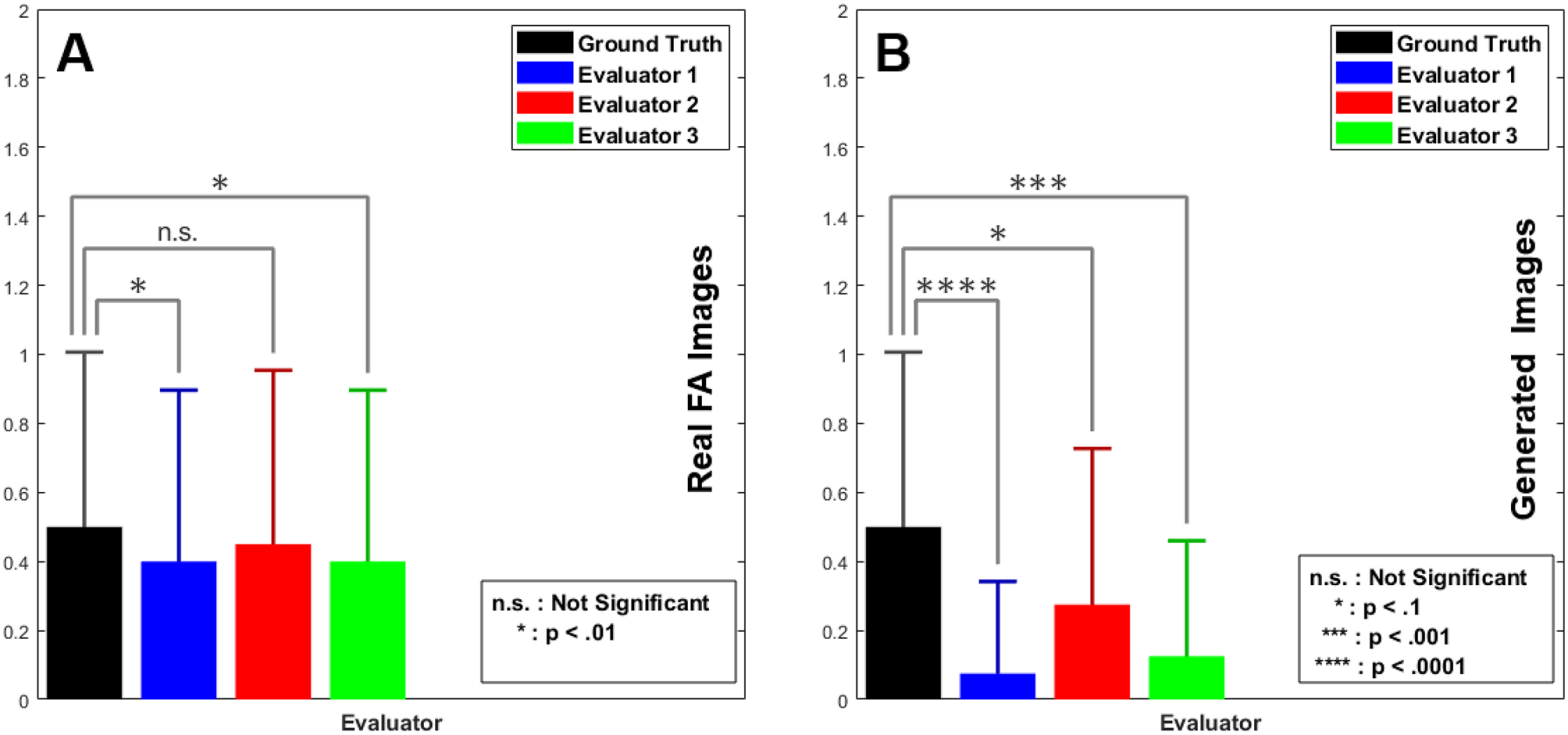
Expert evaluation of real versus generated FA images, given real FA images (**A**) and FA images generated by our proposed approach (**B**). Experts had difficulty distinguishing between real and generated FA images. For real images, two out of three experts had significantly lower correct classification compared to the ground truth (${p} < .1$). Given generated images, all experts had significantly lower correct classification scores compared to the ground truth (${p} < .1$, ${p} < .001$, and ${p} < .0001$).

### Qualitative evaluations

In the next experiment we evaluated the quality of the generated angiograms by asking experts to identify whether a given angiograms is real, from a collection of 40 balanced (50%, 50%) and randomly mixed angiograms. For this experiment, the experts were not told how many of the images are real and how many are not real. The non-disclosed ratio of non-real and real images was a significant design choice for this experiment, as it will allow us to evaluate three metrics: (1) incorrectly identified generated images represent how real the generated images look, (2) correctly labeled real images representing how accurate the experts recognized angiogram salient features, and (3) the confusion metric representing how effective the overall performance of our proposed method was in confusing the expert in the overall experiment. The results are shown in Fig. 10. Given a real FA image, two out of three experts significantly identified fewer real images than the ground truth (Fig. 10A). Given generated angiongrams, all three experts missed significantly more generated angiograms (Fig. 10B). This experiment shows that the generated FA images by our proposed method are virtually indistinguishable from real FA images.

## Discussion

Our study demonstrates that a deep learning generative adversarial network (GAN) can be trained to map anatomical features from different image modalities, i.e. fundus photographs and FA images, onto a shared feature manifold for the purpose of generating one image modality from the other. Once the deep learning network is trained on a training dataset of paired fundus and FA images, it is capable of generating anatomically accurate retinal vasculature in the form of FA images. Our deep learning model was capable of generating accurate and reliable FA images from fundus photographs, even under significant noise, motion blur, and color and contrast manipulations. The most significant aspect of the proposed deep learning architecture is the that it is the first application of deep learning in ophthalmology capable of translating between two different modalities of data. We also utilized a comprehensive data augmentation method to increase the accuracy of our deep learning system without the need for a very large training dataset.

Taking advantage of our study, detailed retinal vascular structures can be created without the need for fluorescein angiogrpahy to avoid its potential side effects. Furthermore, generating vascular images from fundus photography via deep learning generative networks enables a non-invasive, cost effective, easy to use, and low-cost alternative for FA. Bypassing FA protocols by utilizing our proposed deep learning approach has the potential to enable remote monitoring of patients. In addition, generating FA images from fundus photographs does not impose the need for multiple measurements required by OCTA to reconstruct vascular maps over large areas of the retina.

A potential explanation by which the proposed deep learning approach is capable of inferring the retinal vascular structure from fundus images is that a paired set of FA and fundus images of the same eye share the same statistical distributions governing the anatomical structure of the eye from which the images are acquired. Although not visible from fundus images, the light reflects differently from the blood vessels and their neighboring region on the retina. These minute differences, locally and globally, are utilized by our deep learning algorithm to establish shared local and global feature representations from paired FA and fundus images. The trained model is then capable of using these learned shared features to infer the structural statistics of an FA directly from the structural statistics of a given fundus photograph and produce an anatomically accurate FA image counterpart. In fact, this shared feature representation learning is recently used in computer vision to transfer image modalities and styles, e.g. transforming real photographs to art styles of Monet or Van Gogh paintings^60–63^.

The proposed deep learning generative network in our study produces FA images from fundus photographs. This finding has significant clinical applications. Fundus imaging is an easy, low-cost, and non-invasive procedure and is one of the most commonly performed eye procedures, resulting in a very large number of fudnus imaging databases. Moreover, fundus imaging can be done at home from a number of recently introduced portable funduscopes^64–67^. Our study demonstrates the potential for using fundus images acquired from these portable fundus imaging systems to produce reliable and anatomically accurate retina vascular structures. The inferred structural measurements of retinal vasculature may allow clinicians to determine the natural history of retinal vascular changes and clinical outcomes of retinal diseases as previously reported from direct analysis of fundus images^68,69^, but with the accuracy of FA image analysis^70,71^ or even OCTA^72^.

In our work we used a multi-scale conditional deep learning network comprised of two components, a generator block and a discriminator block. The generator block is responsible for sampling a probability distribution function to generate an image. The discriminator block is responsible for deciding whether a given image is a real FA image or a generated one. For training, the entire system undergoes a minimax optimization process^73^, in which the generator tries to produce realistic FA images in such a way that the discriminator cannot correctly label as not real, while the discriminator tries to predict from a pair of real or generated images which one is real, as accurately as possible. To our knowledge, this architecture is the first of its kind to be designed and utilized for ophthalmic applications to generate one modality of images from another. The multi-scale design of the proposed network enables it to perform more accurately with fewer training samples and overcome the data limitations from which the majority of traditional deep learning architecture suffer^28^. Our study and data shows the superior results of our network compared to recent generative networks. Future work would including designing a side network within this generative network capable of mapping anatomical structures and bio markers representative of specific pathologies to establish a latent manifold of pathology feature representations. This latent manifold would be instrumental in predicting future progression of retina vascular disorders much earlier in the disease stage.

More broadly, we demonstrated that deep learning architectures are capable of translating between different ophthalmic image modalities. A similar approach to our architecture could be utilized to establish relationships between ocular anatomical structure measurements, e.g. OCT, MRI, funduscopy, and visual function such as field perimetry, acuity, and color and contrast sensitivities. For example, several physiological assessments such as OCT and funduscopy, and functional measurements such as visual fields assessments of the same eye are usually performed at each clinic visit. A similarly designed network to the proposed architecture can be utilized to map OCT and fundus photographs along with visual fields onto a manifold of shared feature representations. These shared representations can then be utilized to convert visual field progressions to OCT images establishing retinal fiber layer changes in glaucoma patients without the need to perform OCT measurements. In addition, converting available OCT measurements to a more objective representation of the visual field deficits could help evaluate disease progression in a more objective manner without the need to use subjective field perimetry.

**Figure 11:**
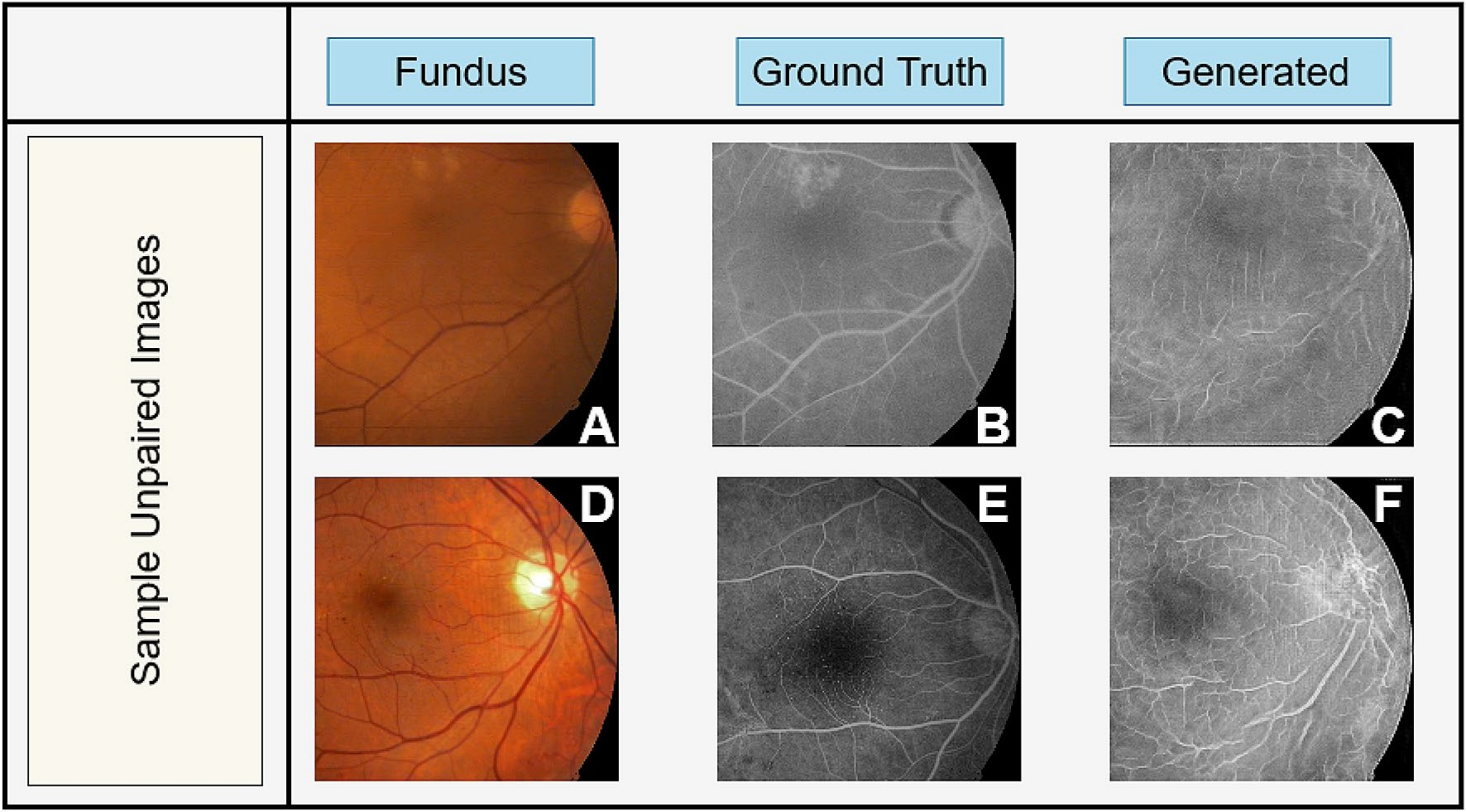
Results of the proposed GAN architecture on unpaired original Fundus images (**A,C**). Compared to the corresponding FA images as ground truth (**B,D**), the proposed architecture will produce un-natural images (**C,E**) on on-paired data.

It is worth noting that the clinical use of generative adversarial networks without sufficient ablation studies on the neural network and its performance in generating images is dangerous. This is due to the GAN potential in producing fake features. General GAN approaches simply sample a random distribution to generate fake images, and therefore are susceptible to producing fake feature. This is particularly the case for GAN architectures designed for an unpaired dataset, as well as in traditional cycleGAN architectures. To avoid this issue we proposed the use of our conditional GAN framework in a paired setup with a hierarchical architecture (Fig. 1A). This is clinically significant, as the proposed method, if applied to unpaired images, will produce unnatural generated FA images. Therefore, the natural FA images are assured to include accurate anatomical features that are trained from paired FA and fundus datasets (Fig. 11).

The limitations of the current study are in the use of a single dataset of paired fundus and FA images, the size of the dataset, and due to data limitations, our inability to perform longitudinal studies of benefits of the proposed method in evaluating disease progression. While this dataset was sufficient for establishing the performance of the proposed deep learning model, further studies are needed on additional paired fundus and FA images to validate the results. In this study we proposed the use of data augmentation and conditional GANs in a multi-scale architecture to overcome the size limitations of the dataset. We anticipate using larger training samples acquired from large datasets will further improve the already establish superior results of our method. Another limitation in the study relates to the lack of information about the phase of the FAG images. The FAG phase information is missing from the current dataset on which the proposed method has been evaluated. In future studies we plan to include this information in our analyses. Finally, future longitudinal studies could prove the benefits of utilizing the proposed deep learning method in generating FA images from fundus photographs for regularly monitoring retinal vascular disease progression in ways not possible before while avoiding costs and side effects associated with FA.

In conclusion, we demonstrated that a deep learning based generative adversarial network (GAN) is capable of producing FA images from single fundus photographs alone, that are virtually indistinguishable from real FA images. This approach can be used on any existing fundus photograph dataset or could be integrated into funduscopy system to produce FA images along with the fundus photographs. Although our proposed framework provides an unrivaled way for the translation of images from one domain to the other, this study is designed as a proof-of-concept framework to demonstrate the technical and computational viability of performing image domain transformation to provide adjunctive information in the absence of FA modalities. Future studies are needed to validate how diagnostic capabilities may be improved by utilizing our framework in the absence of a FA test results.

## Methods

This study utilizes publicly available and de-identified paired fluorescein angiogram and fundus photographs from the Isfahan University of Medical Sciences Persian Eye Clinic (Feiz Hospital)^55^. The study has been approved by the University of Nevada, Reno Institutional Review Board for the use of retrospective de-identified data and all methods were performed in accordance with the relevant guidelines and regulations. This study only uses anonymized and de-unidentified data. However, informed consent was obtained from all subjects, who were over the age of 18, as a part of the original study. Retinal images ($576\times 720$ pixels) were collected and include 30 normal stage and 40 abnormal stages.

### Deep learning conditional generative adversarial network

This study proposes a new conditional generative adversarial network (GAN) comprising of a novel residual block^44,45^ for producing realistic FA from retinal fundus images. We use two generators ($G_{fine}$ and $G_{coarse}$) in the proposed network, as illustrated in Fig. 1A. The generator $G_{fine}$ synthesizes fine angiograms from fundus images by learning local information, including retinal venules, arterioles, hemorrhages, exudates, and microaneurysms. On the other hand, the generator $G_{coarse}$ tries to extract and preserve global information, such as the structures of the macula, optic disc, color, contrast and brightness, while producing coarse angiograms. The generator $G_{fine}$ takes input images of size $512\times 512$ and produces output images with the same resolution. Similarly, the generator $G_{coarse}$ network takes an image with half the size ($256\times 256$) and outputs an image of the same size as the input. In addition, the $G_{coarse}$ outputs a feature vector of the size $256\times 256 \times 64$ that is eventually added with one of the intermediate layers of $G_{fine}$. These hybrid generators are quite powerful for sharing local and global information between multiple architectures as seen in^50,52,74^. Both generators use convolution layers for downsampling and transposed convolution layers for upsampling. It should be noted that $G_{coarse}$ is downsampled twice ($\times 2$) before being upsampled twice again with transposed convolution. In both the generators, the proposed residual blocks are used after the last downsampling operation and before the first upsampling operations as illustrated in Fig. 1B. On the other hand, in $G_{fine}$, downsampling takes place once with necessary convolution layer, followed by adding the feature vector, repetition of residual blocks and then upsampling to get fine angiography image. All convolution and transposed convolution operation are followed by Batch-Normalization^75^ and Leaky-ReLU activations. To train these generators, we start with $G_{coarse}$ by batch-training it on random samples once and then we train the $G_{fine}$ once with a new set of random samples. During this time, the discriminator’s weights are frozen. Lastly, we jointly fine-tune all the discriminator and generators together to train the GAN.

### Multi-scale PatchGAN as discriminator

For synthesizing fluorescein angiography images, GAN discriminators need to adapt to coarse and fine generated images for distinguishing between real and generated images. To alleviate this problem, we either need a deeper architecture or, a kernel with wider receptive field. Both these solutions result in over fitting and increase the number of parameters. Additionally, a large amount of processing power will be required for computing all the parameters. To address this issue, we exploit the idea of using two Markovian discriminators, first introduced in a technique called PatchGAN^76^. This technique takes input from different scales as previously seen in^50,52^. We use four discriminators that have a similar network structure but operate at different image scales. Particularly, we downsample the real and generated angiograms by a factor of 2 using the Lanczos sampling^77^ to create an image pyramid of three scales (original and $2\times$downsampled and $4\times$downsampled). We group the four discriminators into two, $D_{fine}=[D1_{fine},D2_{fine}]$ and $D_{coarse}=[D1_{coarse},D2_{coarse}]$ as seen in Fig. 1A. The discriminators are then trained to distinguish between real and generated angiography images at the three distinct resolutions respectively.

The outputs of the PatchGAN for $D_{fine}$ are $64\times 64$ and $32\times 32$ and for $D_{coarse}$ are $32\times 32$ and $16\times 16$. With the given discriminators, the loss function can be formulated as given in Eq. 1. It is a multi-task problem of maximizing the loss of the discriminators while minimizing the loss of the generators.

1 $$\begin{aligned} \min \limits _{G_{fine},G_{coarse}} \max \limits _{D_{fine},D_{coarse}} {\mathscr {L}}_{cGAN}(G_{fine},G_{coarse}, D_{fine},D_{coarse}) \end{aligned}$$

Despite discriminators having similar network structure, the one that learns features at a lower resolution has wider receptive fields. It tries to extract and retain more global features such as macula, optic disc, color and brightness to better distinguish real images. In contrast, the discriminator that learns feature at original resolution dictates the generator to produce fine features such as retinal veins, arteries, and exudates. By doing this we combine feature information of global and local scale while training the generators independently with their paired multi-scale discriminators.

### Weighted objective function and adversarial loss

We use LSGAN^78^ to train our conditional GAN. The objective function for our conditional GAN is given in Eq. 2.

2 $$\begin{aligned} {\mathscr {L}}_{cGAN}(G,D) = {\mathbb {E}}_{x,y} \big [\ (D(x,y) -1)^2 \big ]\ + {\mathbb {E}}_{x} \big [\ (D(x,G(x)+1))^2 \big ]\ \end{aligned}$$

where the discriminators are first trained on the real fundus, *x* and real angiography image, *y* and then trained on the the real fundus, *x* and generated angiography image, *G*(*x*). We start with training the discriminators $D_{fine}$ and $D_{coarse}$ for couple of iterations on random batches of images. Next, we train the $G_{coarse}$ while keeping the weights of the discriminators frozen. We then train the the $G_{fine}$ on a batch of random samples in a similar fashion. We use Mean-Squared-Error (MSE) for calculating the individual loss of the generators as shown in Eq. 3.

3 $$\begin{aligned} {\mathscr {L}}_{L2}(G) = {\mathbb {E}}_{x,y} \Vert G(x) - y \Vert ^2 \end{aligned}$$

where ${\mathscr {L}}_{L2}$ is the reconstruction loss for a real angiogram, *y*, given a generated angiogram, *G*(*x*). We use this loss for both $G_{fine}$ and $G_{coarse}$ so that the model can generate high quality angiograms at different scales. From Eqs. 2 and 3 we can formulate our final objective function as given in Eq. 4.

4 $$\begin{aligned} \min \limits _{G_{fine},G_{coarse}} \max \limits _{D_{fine},D_{coarse}} {\mathscr {L}}_{cGAN}(G_{fine},G_{coarse}, D_{fine},D_{coarse}) + \lambda \big [\ {\mathscr {L}}_{L2}(G_{fine}) + {\mathscr {L}}_{L2}(G_{coarse})\big ]\ \end{aligned}$$

Here, $\lambda$ dictates either to prioritize the discriminators or the generators. For our architecture, more weight is given to the reconstruction loss of the generators and thus we pick a large $\lambda$ value.

### Computational resources

The computational resources used for this study included an Alienware Aurora R9 Gaming Desktop, with Intel Core i7-9700 central processing unite (CPU), 16GB Memory, and an NVIDIA GeForce RTX 2080 SUPER graphics processing unit (GPU). The code was written in python with Keras wrapper for TensorFlow.

## Data Availability

The dataset analyzed for this study is comprised of de-identified fundus and FA images publicly available from the Isfahan University of Medical Sciences^79^.
